# Prognostic score in patients with recurrent or metastatic carcinoma of the head and neck treated with cetuximab and chemotherapy

**DOI:** 10.1371/journal.pone.0180995

**Published:** 2017-07-07

**Authors:** Teresa Magnes, Thomas Melchardt, Lukas Weiss, Christof Mittermair, Daniel Neureiter, Eckhard Klieser, Simon Gampenrieder, Gerhard Moser, Alexander Gaggl, Richard Greil, Alexander Egle

**Affiliations:** 1 IIIrd Medical Department at the Paracelsus Medical University, Salzburg, Austria; 2 Salzburg Cancer Research Institute, Salzburg, Austria; 3 Cancer Cluster Salzburg, Salzburg, Austria; 4 Institute of Pathology at the Paracelsus Medical University, Salzburg, Austria; 5 Department of Otorhinolaryngology, Head and Neck Surgery, Paracelsus Medical University, Salzburg, Austria; 6 Department of Oral and Maxillofacial Surgery, Paracelsus Medical University, Salzburg, Austria; University of Cincinnati College of Medicine, UNITED STATES

## Abstract

Despite modern treatment approaches, survival of patients with recurrent or metastatic squamous cell carcinoma of the head and neck (SCCHN) remains low and it is difficult to identify patients who derive optimal benefit from treatment. We therefore analyzed which commonly available laboratory and clinical parameters may help improve the prognostication in this patient group. This retrospective monocenter analysis includes 128 patients with recurrent or metastatic SCCHN treated with cetuximab alone or in combination with polychemotherapy as first line therapy. Factors with independent prognostic power in the multivariate analysis were used to build up a score separating patient groups with different survival. Patients had a median age of 61 years and 103 patients were treated with polychemotherapy plus cetuximab. An ECOG score above 1, high CRP and leukocyte levels, less intensive treatment and a time below 12 months from primary diagnosis to relapse remained as independent negative prognostic factors in multivariate analysis.

Patients with 0 to 1 risk factors had a median OS of 13.6 months compared to a median OS of less than one month for patients 4 to 5 risk factors (*p*<0.001). This study identifies 5 clinical and serum values that influence survival of patients with recurrent or metastatic SCCHN treated with cetuximab. By combining these factors to create a score for OS, it is possible to distinguish a group of patients with significantly improved survival and define those most likely to have no benefit from cetuximab treatment.

## Introduction

Approximately 650.000 new cases of squamous cell carcinoma of the head and neck (SCCHN) are diagnosed each year worldwide and despite modern treatment approaches more than a third of patients with advanced disease experience relapse not suitable for therapy with curative intent.[1–5] The standard of care for these patients was chemotherapy including platinum compounds, fluorouracil, methotrexate or taxanes for many years with prognosis still grim. There is evidence for the activity of monotherapy with the anti-epidermal growth factor receptor (EGFR) antibody cetuximab in recurrent or metastatic SCCHN with a disease control rate of 46% in platinum resistant patients and acceptable toxicity rates.[6] Platinum-based chemotherapy in combination with cetuximab has been advocated as the standard first line therapy for patients without curative treatment options in international treatment guidelines due to the results of a phase III trial published in 2008.[7] Only very recently, progress has been made with the introduction of checkpoint inhibitors in this population.[8, 9]

Several prognostic biomarkers such as human papilloma virus (HPV) infection, p53 and chemokines have been proposed in SCCHN.[10–14] However, standardized tools to identify patients with a low probability of benefit from treatment are missing and molecular markers are often expensive and not routinely available in clinical practice.

Nevertheless, a complete blood count and inflammation markers such as c-reactive protein (CRP) are usually available in each patient before the start of chemotherapy. The negative prognostic influence of markers of chronic inflammation was described in different tumor entities such as renal cancer, non hodgkin lymphoma and pancreatic cancer but the impact of CRP, leukocytes and hemoglobin levels on the survival of patients with recurrent or metastatic SCCHN is not known.[15–19]

We set out to define which laboratory and clinical parameters may be suitable to help improve the prognostication in first line palliative treatment for patients with SCCHN.

## Materials and methods

### Patients

All patients included in this retrospective analysis were diagnosed with recurrent or metastatic SCCHN and were considered incurable by a multidisciplinary tumor board at our cancer center. Patients were treated with cetuximab alone or in combination with chemotherapy as first line palliative treatment between September 2006 and March 2016 at the 3^rd^ Medical Department of the Paracelsus Medical University Salzburg. If patients were considered suitable for combination therapy, they received cisplatin or carboplatin, fluorouracil and cetuximab, in analogy to the study by Vermorken et al published in 2008.[7] Other patients were treated with single agent chemotherapy and cetuximab or cetuximab alone.

Patient characteristics such as age, performance score, nutritional status, substance abuse and tumor characteristics were retrospectively analyzed by chart-based review.

Immunohistochemistry for p16 expression with a staining cutoff of 10% was used as a surrogate parameter for HPV infection. The HPV status was available for the majority (74.7%) of patients with a primary tumor of the oropharynx, the oral cavity or the larynx. Patients with missing p16 staining were classified as “not available”.

To evaluate the prognostic role of routinely obtained serum values, complete blood count, CRP and liver enzymes were analyzed within two weeks before the start of therapy. Progression free Survival (PFS) was defined as the time from the start of palliative treatment to progression or death from any cause and OS was calculated from the start of treatment to death from any cause. The last update of the database was made on the 25^th^ of July 2016 and no patients were lost to follow up.

This analysis was approved the Ethics Committee of the provincial of Salzburg Austria (415-EP/73/662-2016) and all patients gave their written informed consent.

### Statistical analyses

All statistical analyses were performed using IBM® SPSS® statistics software, version 21. Mann-Whitney-U-test and Pearson’s chi-square test were used for univariate analyses, where appropriate. Survival was estimated using Kaplan-Meier curve analysis, with statistical comparisons using the log-rank statistic. A two-tailed significance level of 0.05 was considered statistically significant. Cutoff values for serum markers and age of our patients were determined by Receiver Operating Characteristic (ROC) calculation and Youden Index analysis for OS. Only factors that had a significant influence on OS in univariate analysis were included in multivariate Cox-regression analysis. Independent prognostic factors were used to build a score to predict OS.

## Results

### Patient characteristics

Between 2006 and 2016, 128 patients were treated with cetuximab as first line palliative therapy for recurrent or metastatic squamous cell carcinoma of the head and neck. 103 (80.5%) of these patients received combination therapy consisting of a platinum compound, fluorouracil plus cetuximab. Their clinical characteristics are shown in Table 1. Briefly, the median age of all patients at the start of palliative treatment was 60.5 years and the majority (84.4%) were male. As would be expected, patients receiving polychemotherapy plus cetuximab had a better performance score and a higher body mass index (BMI) than patients receiving less intensive therapy (74.8% of patients with ECOG 0 or 1 versus (vs.) 44.0%, *p* = 0.003 and median BMI of 21.0 vs. 18.9, *p* = 0.015). The polychemotherapy plus cetuximab group also had a higher tumor stage at the time of primary diagnosis (93.9% AJCC stage 3 or 4 vs. 77.3%, *p = 0*.*015*). Furthermore, more patients treated with combination therapy had previously received radiotherapy as part of curative treatment for local disease (82.5% in the polychemotherapy plus cetuximab group vs. 56.0% in the less intensive therapy group, *p* = 0.004). More than half of all patients (66.4%) had already received systemic therapy as part of curative treatment before the start of palliative therapy. The smoking status was known for the majority (75.0%) of the patients. Out of these, 85.4% were smokers. Furthermore, we knew of alcohol abuse in 33.6% of our patients. Five out of 17 patients (29.4%) with oropharynx cancer, 8 out of 22 patients (36.4%) with larynx cancer and 5 out of 29 patients (17.2%) with a tumor of the oral cavity had p16 positive disease.

**Table 1:** Characteristics of patients with recurrent or metastatic head and neck cancer treated with cetuximab.

	all patients	polychemotherapy plus cetuximab	less intensive therapy	*p-value*
	(*n = 128*)	(*n = 103*)	(*n = 25*)	
age at primary diagnosis				
median (years)	58.0	58.0	60.0	*0*.*154*^*1*^
range	32–90	32–74	44–90	* *
age at palliative treatment start				
median (years)	60.5	60.0	67.0	*0*.*081*^*1*^
range	35–90	35–75	46–90	* *
gender				* *
male	84.4% (108)	87.4% (90)	72.0% (18)	*0*.*057*^*2*^
female	15.6% (20)	12.6% (13)	28% (7)	
primary tumor site				*0*.*264*^*2*^
oral cavity	33.6% (43)	30.1% (31)	48.0% (12)	* *
p16 positive	11.6% (5)	5 (16.1%)	n.a.	* *
p16 negative	55.8% (24)	24 (77.4%)	n.a.	* *
p16 unknown	32.6% (14)	2 (6.5%)	n.a.	* *
oropharynx	18.0% (23)	16.5% (17)	24.0% (6)	* *
p16 positive	21.7% (5)	29.4% (5)	n.a.	* *
p16 negative	52.2% (12)	70.6% (12)	n.a.	* *
p16 unknown	26.1% (6)	0% (0)	n.a.	* *
larynx	19.5% (25)	23.3% (24)	4.0% (1)	* *
p16 positive	32.0% (8)	33.3% (8)	n.a.	* *
p16 negative	56.0% (14)	58.3% (14)	n.a.	* *
p16 unknown	12.0% (3)	8.3% (2)	n.a.	* *
hypopharynx	12.5% (16)	13.6% (14)	8.0% (2)	* *
other	16.4% (21)	16.5% (17)	16.0% (4)	* *
AJCC stage at primary diagnosis				*0*.*015*^*2*^
stage 1–2	5.6% (11)	5.8% (6)	20.0% (5)	* *
stage 3–4	85.2% (109)	89.3% (92)	68.0% (17)	* *
ECOG score 0–1 at palliative treatment start	68.8% (88)	74.8% (77)	44.0% (11)	*0*.*003*^*2*^
BMI				* *
median	20.8	21.0	18.9	*0*.*015*^*1*^
range	14.0–32.45	15.0–32.5	14.0–27.3	* *
prior therapy				* *
prior radiotherapy	77.3% (99)	82.5% (85)	56.0% (14)	*0*.*004*^*2*^
prior chemotherapy	51.6% (66)	55.3% (57)	36.0% (9)	*0*.*083*^*2*^
prior cetuximab therapy	20.3% (26)	19.4% (20)	24.0% (6)	*0*.*609*^*2*^
distant metastasis	50.8% (65)	47.6% (49)	64.0% (16)	*0*.*141*^*2*^
second line palliative therapy				*0*.*181*^*2*^
yes	51.6% (66)	54.4% (56)	40.0% (10)	* *
no	47.7% (61)	44.7% (46)	60.0% (15)	* *
palliative radiotherapy	31.2% (40)	25.2% (26)	56.0% (14)	*0*.*003*^*2*^
palliative tumor resection	5.5% (7)	5.8% (6)	4.0% (1)	*0*.*719*^*2*^
cigarette abuse				*0*.*693*^*2*^
yes	64.1% (82)	66.0% (68)	56% (14)	* *
no	10.9% (14)	10.7% (11)	12% (3)	* *
second malignancy	19.5% (25)	19.4% (20)	20.0% (5)	*0*.*947*^*2*^

Note. Some values don’t add up to 100% due to missing data, ^1^ = Mann-Whitney-U-Test, ^2^ = Pearson’s Chi Square Test, n.a. = not available, AJCC = American Joint Committee on Cancer, BMI = body mass index

### Clinical outcome

**Fig 1:**
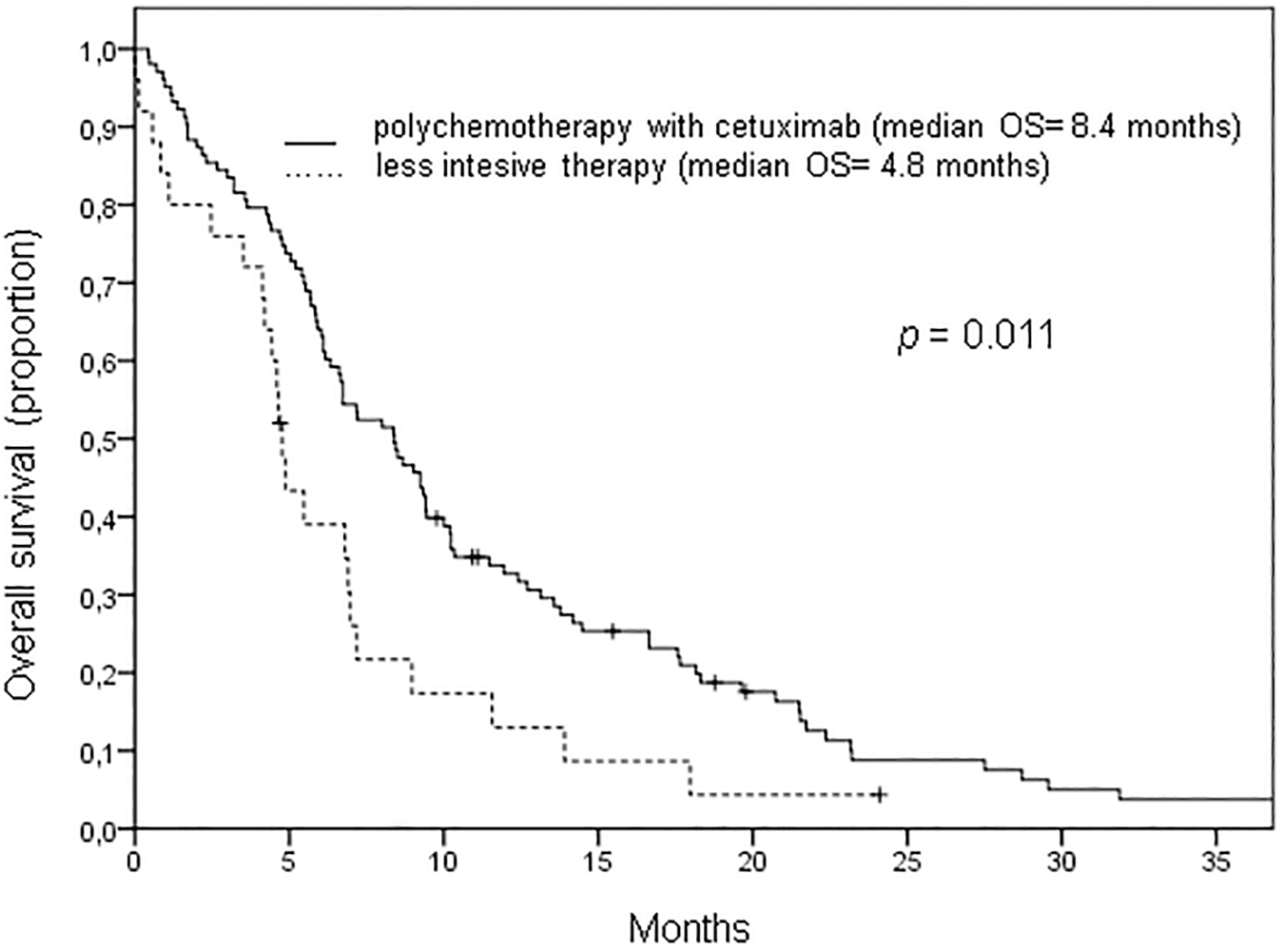
Overall survival of cetuximab treated patients with recurrent or metastatic head and neck cancer. Median overall survival (OS) of patients treated with polchemotherapy plus cetuximab was significantly longer compared to patients treated with less intensive therapy (median OS: 8.4 months compared to 4.8 months, *p = 0*.*011*).

**Fig 2:**
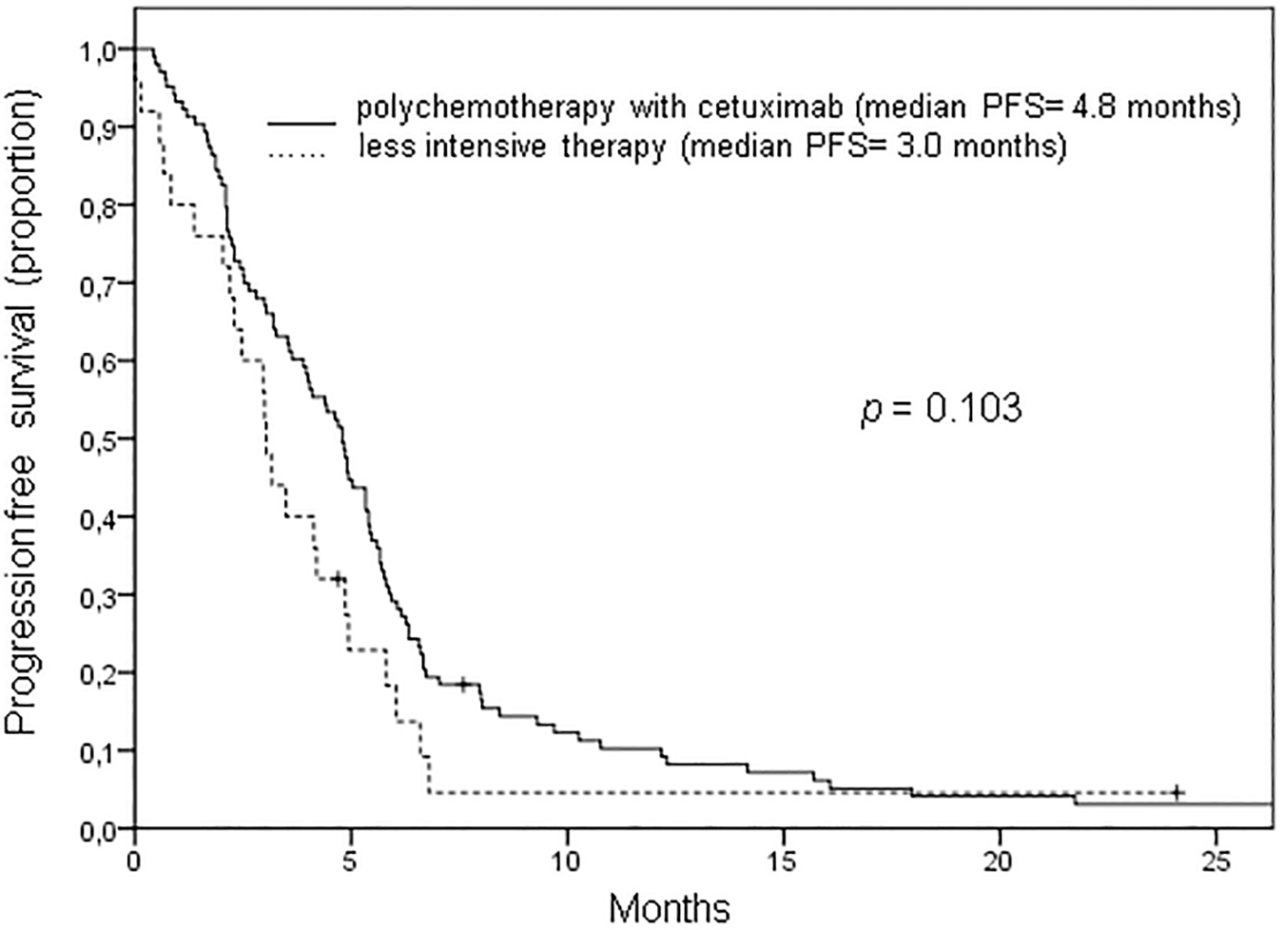
Progression free survival of cetuximab treated patients with recurrent or metastatic head and neck cancer. Median progression free survival (PFS) of patients treated polychemotherapy plus cetuximab was 4.8 months compared to a median PFS of 3.0 months for patients treated with less therapy (*p = 0*.*103*).

Median PFS and OS of all patients from the start of palliative systemic therapy were 4.4 and 6.9 months respectively. Acknowledging the differences in patient characteristics, OS was significantly better for patients receiving polychemotherapy plus cetuximab than for patients treated with a less intensive regimen (8.4 vs. 4.8 months, *p = 0*.*011*, Fig 1). However, differences in PFS between these two groups of patients did not reach the level of significance (4.8 vs. 3.0 months, *p* = 0.103, Fig 2). Analyzing only patients with an ECOG score of 0 to 1 and a systemic therapy free interval of 6 months before start of palliative treatment similar to the inclusion criteria of the phase III study by Vermorken et al [7], the median PFS and OS were 5.7 months and 13.1 respectively.

There was no statistically significant difference between survival of patients with p16 positive tumors compared to patients with p16 negative tumors in this cohort (median OS 9.2 vs. 8.0 months, *p =* 0.798; median PFS 4.0 vs. 4.8 months, *p =* 0.755).

### Prognostic factors for OS

Cutoff values for CRP, leukocytes, hemoglobin, thrombocytes, gamma-glutamyl transferase and age determined to be optimal to discriminate the OS were as follows: 8.5 mg/L, 9.25 G/L, 11.25 g/dl, 281 G/L and 30.5 U/L, 60.5years respectively. The median duration of time between primary diagnosis and the start of palliative cetuximab treatment was 12.1 months. A chemotherapy free interval of at least 6 months was an inclusion criterion of the EXTREME trial. With the addition of surgery and/or radiotherapy this also results in a time span of about a year between primary diagnosis and recurrence. Therefore, 12 months was considered as a clinically meaningful cutoff for early disease recurrence. The serum markers as well as clinical characteristics were tested in univariate analyses for OS. ECOG > 1, high CRP or leukocyte levels, the treatment regimen and a time below 12 months from primary diagnosis to relapse not amenable to curative therapy remained as independent prognostic factors in multivariate Cox-regression analysis (see Table 2).

**Table 2:** Prognostic factors for overall survival of patients with recurrent or metastatic head and neck cancer.

		univariate Analysis			multivariate Analysis		
Variable		Hazard Ratio(95% CI)	*p*^1^	*n*	Hazard Ratio(95% CI)	*p*^1^	*n*
**ECOG score**	high (2–3) vs. low (0–1)	2.175(1.463–3.235)	<0.001	128	2.048(1.319–3.179)	0.001	122
**c-reactive protein**	above 8.5 mg/L vs. below	1.955(1.296–2.950)	0.001	123	1.651(1.058–2.575)	0.027	122
**leucocytes**	above 9.25G/L vs. below	2.351(1.564–3.533)	<0.001	126	2.224(1.416–3.495)	0.001	122
**time from primary diagnosis to palliative therapy**	below 12 months vs. above 12 months	1.561(1.080–2.256)	0.018	128	1.830(1.185–2.824)	0.006	122
**thrombocytes**	above 281 G/L vs. below	1.725(1.189–2.503)	0.004	126	1.215(0.784–1.881)	0.383	122
**first line palliative treatment**	less intensive therapy vs. polychemotherapy plus cetuximab	1.811(1.140–2.878)	0.012	128	2.560(1.510–4.341)	<0.001	122
**prior radiotherapy**	yes vs. no	0.590(0.384–0.905)	0.016	128	1.039(0.621–1.738)	0.884	122
**hemoglobin**	above 11.25g/dl vs. below	0.800(0.547–1.169)	0.249	126	n.a.		
**gamma-glutamyl transferase**	above 30.5 U/L vs. below	1.315(0.888–1.949)	0.172	124	n.a.		
**age**	above 65 vs. below	1.219(0.834–1.781)	0.307	128	n.a.* *		
**prior chemotherapy**	yes vs. no	0.950(0.660–1.366)	0.780	128	n.a.		
**prior cetuximab**	yes vs. no	0.933(0.584–1.489)	0.770	128	n.a.		
**tumor site**	other vs. oropharynx	1.138(0.695–1.862)	0.608	128	n.a.		
**grading**	G3 vs. G2/G1	0.843(0.569–1.250)	0.396	124	n.a.		
**distant metastasis**	yes vs. no	1.002(0.697–1.440)	0.993	128	n.a.		

^1^ = cox regression analysis, CI = confidence interval, vs. = versus, mg/L = milligram per liter, G/L = giga per liter, g/dl = gram per deciliter, U/L = unit per liter, n.a. = not available

**Fig 3:**
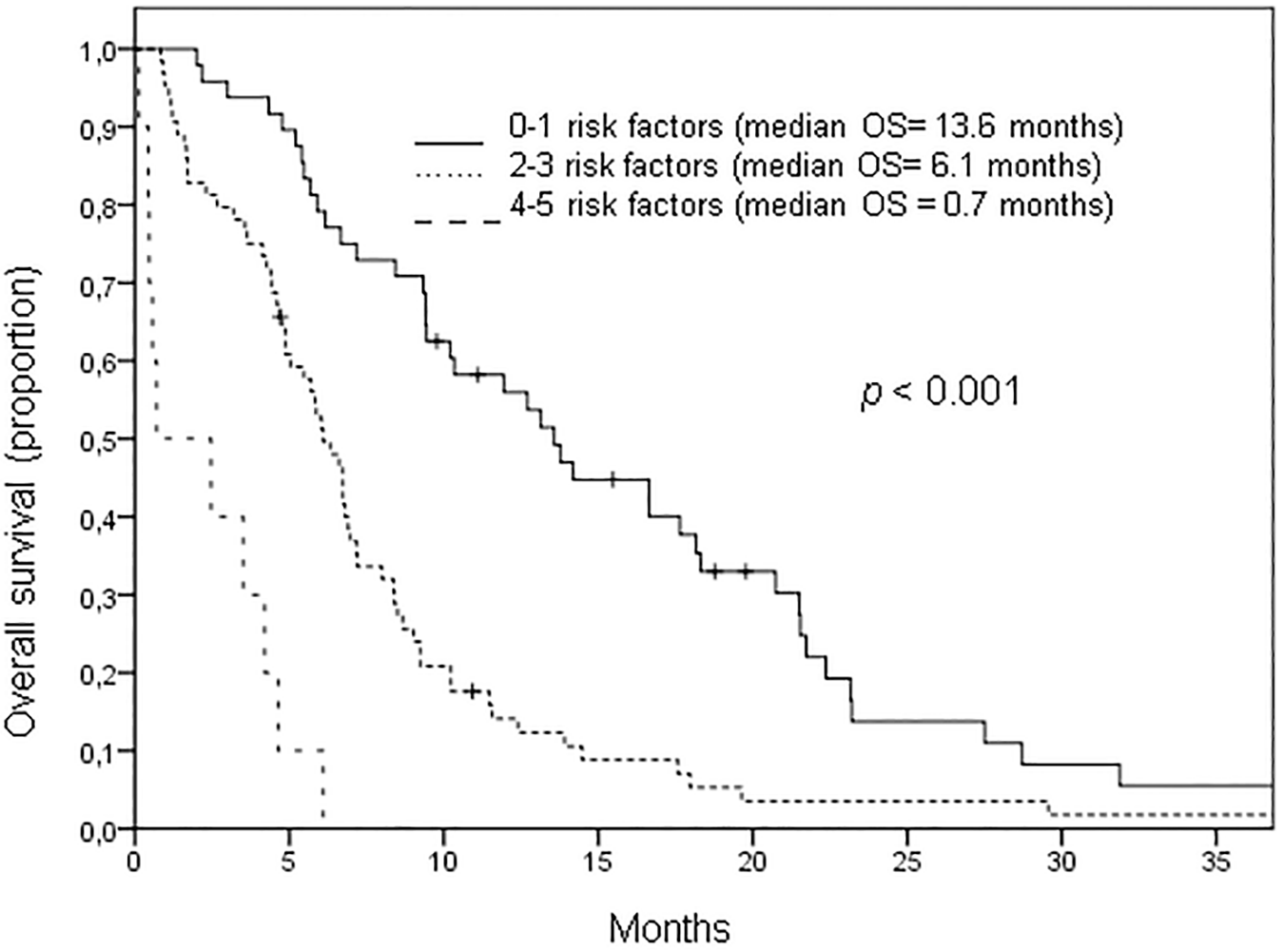
Overall survival score for all patients with recurrent or metastatic head and neck cancer. 103 patients were treated with polychemotherapy and cetuximab and 25 patients were treated with cetuximab alone or in combination with single agent chemotherapy. An ECOG score above 1, high CRP and leukocyte levels, less intensive treatment and a time below 12 months from primary diagnosis to relapse were found to be independent prognostic factors for overall survival (OS). Patients with 0 to 1 risk factors had a median OS of 13.6 months compared to 6.1 and 0.7 months for patients with 2 to 3 and 4 to 5 risk factors, respectively (*p<0*.*001*).

By attributing one point for each of these 5 values, a prognostic score was generated for all patients. The median OS was 21.5, 12.7, 6.8, 4.9, 0.7 and 0.6 months for patients with 0, 1, 2, 3, 4 or 5 risk factors respectively (*p*<0.001). Patients with 0 to 1 risk factors, 2 to 3 and 4 to 5 risk factors were grouped and the median OS was 13.6, 6.1 and 0.7 months respectively (*p*<0.001, see Fig 3). The same score can also be applied for median PFS of all patients (median PFS 0–1 risk factors: 4.9months, 2–3 risk factors: 3.6months, 4–5 risk factors: 0.7 months, *p*<0.001).

**Fig 4:**
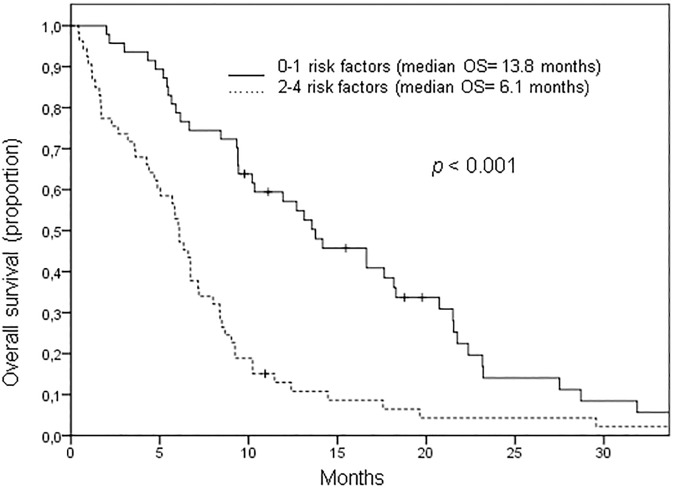
Overall survival score for patients treated with polychemotherapy plus cetuximab. 103 patients were treated with polychemotherapy and cetuximab. Among these patients, an ECOG score above 1, high CRP and leukocyte levels and a time below 12 months from primary diagnosis to relapse were found to be independent prognostic factors for overall survival (OS). Patients with 0 to 1 risk factors had a median OS of 13.8 months compared to 6.1 months for those with 2 to 4 risk factors (*p<0*.*001*).

Median OS of patients treated with polychemotherapy and cetuximab and 0 to 1 risk factors was 13.8 months compared to 6.1 months for those with 2 to 4 risk factors (*p*<0.001, see Fig 4).

## Discussion

This report of patients treated with cetuximab alone or as part of first line palliative therapy, represents one of the largest cohorts treated outside of a clinical study.

Despite the fact that SCCHN is the sixth most common cancer type, literature on palliative patient cohorts and prognostic factors is scarce.[1] Systemic treatment of patients with SCCHN in Salzburg and surrounding regions is centralized at our department due to the characteristics of the Austrian health care system, where there are no oncologic practitioners and radiation oncologist in private practice. This means that our analysis is likely to be less biased regarding insurance status or other socioeconomic factors than international randomized clinical trials.

The survival data for patients receiving a platinum compound, fluorouracil and cetuximab with a median PFS of 4.8 months and a median OS of 8.4 months are somewhat lower in this study than in the phase III clinical trial by Vermorken et al where the median PFS and OS were 5.6 and 10.1 months, respectively. This may reflect differences in patient characteristics and comorbidities of cohorts seen in daily practice compared to those treated in clinical studies. Importantly, patients with a Karnofsky performance score lower than 70 and patients that had received chemotherapy as part of treatment for locally advanced disease during the last 6 months were primarily excluded from the phase III trial by Vermorken et al. If more stringent inclusion criteria regarding ECOG and systemic treatment free interval of at least 6 months are applied to our patients, results are in line with previously published data.[7, 20–22] Furthermore, patients with a Karnofsky score below 80 did not benefit from the addition of cetuximab to chemotherapy in the study published by Vermorken et al.[7] This suggests, that patient selection is very important to avoid intensive and potentially toxic treatment in patients with little chance for benefit. However, a standardized tool to identify these patients was not available so far. We therefore set out to find prognostic variables that would aid such a selection.

The performance score is a known prognostic factor in patients with recurrent or metastatic SCCHN treated with EGFR-antibodies in combination with chemotherapy or chemotherapy alone.[7, 23, 24] Furthermore, in the SPECTRUM trial, in which patients were treated with cisplatin, fluoruracil and panitumuab, weight loss of more than 5% during the last 6 months and prior platinum chemotherapy were identified as independent negative prognostic factors. However, the presence of distant metastasis does not seem to influence OS of patients with recurrent or metastatic SCCHN.[7, 23] Argiris et al described the influence of tumor differentiation and tumor site on the survival of patients treated with cisplatin doublets. Well or moderate tumor differentiation and a primary tumor in the oral cavity or the hypopharynx were determined as unfavorable prognostic factors for OS.[24]

In this study, an ECOG score above 1, the palliative treatment regimen and a time below 12 months from primary diagnosis to relapse not amenable to curative therapy were determined as independent prognostic factors for OS. However, the tumor site, age, presence of distant metastasis and prior therapy did not influence survival.

The critical role of inflammation on tumor progression has been described in different entities and there are several reports on the unfavorable influence of increased CRP levels and other markers of chronic inflammation on the prognosis of different types of tumors such as prostate cancer, ovarian cancer and diffuse large B-cell lymphoma.[15–19] Published data also show that elevated CRP values and neutrophil to lymphocyte ratios have adverse effects on the survival of patients with localized head and neck cancer, treated with curative intent.[25–30] The calculated cutoff values for leukocytes and CRP in this study were 9.25 G/L and 8.5 mg/L respectively, which is close to the upper normal limits. High CRP and high leukocytes, which may reflect inflammation caused by the tumor as well as individual comorbidities, were also poor prognostic factors for patients in this cohort.

By combining all 5 independent prognostic factors found in this study to build a score for OS, it is possible to distinguish a group of patients with significantly improved survival. Patients with good performance score, treatment with polychemotherapy and cetuximab low CRP and leukocyte values and longer time between primary diagnosis and relapse not amenable for curative therapies (0 risk factors) had a favorable median OS of 21.5 months compared to a median OS of less than a month for patients with 4 or 5 risk factors. The median OS for patients with 0 or 1 risk factors is 13.6 months which is 4 months longer than the median OS of patients receiving a platinum compound, fluorouracil and cetuximab in the EXTREME study, which mainly selected patients with a good performance status and normal organ function.[7] The same score was also applicable to the subgroup of patients treated with polychemotherapy plus cetuximab in this cohort. To the best of our knowledge, this is the first prognostic score for cetuximab treated patients with SCCHN.

Our work has several limitations. The smoking status is known in the majority of patients. However, detailed information on alcohol abuse would have been desirable but was not available for all patients due to the lack of a standard assessment tool used in clinical practice. However, immunohistochemistry for p16 expression was available for a large number of patients and while it might overestimate the rate of HPV-positivity especially in non-oropharynx cancer, p16 is similarly associated with improved survival.[10, 31, 32]

As this is a retrospective analysis of a large cohort of patients treated at a single institution, we are currently not able to validate our results in a second group of patients due to the scarcity of such cohorts. However, we would still like to share our findings with the scientific community, as they could be helpful for the guidance and management of patients seen in daily practice.

### Conclusions

In summary, we were able to find 5 independent clinical and serum factors that influence survival of patients with recurrent or metastatic SCCHN treated with cetuximab alone or as part of first line palliative therapy. By combining the factors to create a score that predicts OS, it is possible to distinguish a group of patients with favorable prognosis.

## References

[1] Argiris A, Karamouzis MV, Raben D, Ferris RL. Head and neck cancer. Lancet. 2008;371(9625):1695–709. Epub 2008/05/20. doi: 10.1016/S0140-6736(08)60728-X . 18486742 10.1016/S0140-6736(08)60728-X PMC7720415

[2] Vermorken JB, Remenar E, van Herpen C, Gorlia T, Mesia R, Degardin M, et al Cisplatin, fluorouracil, and docetaxel in unresectable head and neck cancer. N Engl J Med. 2007;357(17):1695–704. Epub 2007/10/26. doi: 10.1056/NEJMoa071028 . 17960012 10.1056/NEJMoa071028

[3] Bonner JA, Harari PM, Giralt J, Cohen RB, Jones CU, Sur RK, et al Radiotherapy plus cetuximab for locoregionally advanced head and neck cancer: 5-year survival data from a phase 3 randomised trial, and relation between cetuximab-induced rash and survival. The Lancet Oncology. 2010;11(1):21–8. Epub 2009/11/10. doi: 10.1016/S1470-2045(09)70311-0 . 19897418 10.1016/S1470-2045(09)70311-0

[4] Posner MR, Hershock DM, Blajman CR, Mickiewicz E, Winquist E, Gorbounova V, et al Cisplatin and fluorouracil alone or with docetaxel in head and neck cancer. N Engl J Med. 2007;357(17):1705–15. Epub 2007/10/26. doi: 10.1056/NEJMoa070956 . 17960013 10.1056/NEJMoa070956

[5] Keil F, Selzer E, Berghold A, Reinisch S, Kapp KS, De Vries A, et al Induction chemotherapy with docetaxel, cisplatin and 5-fluorouracil followed by radiotherapy with cetuximab for locally advanced squamous cell carcinoma of the head and neck. Eur J Cancer. 2013;49(2):352–9. Epub 2012/09/18. doi: 10.1016/j.ejca.2012.08.004 . 22981499 10.1016/j.ejca.2012.08.004

[6] Vermorken JB, Trigo J, Hitt R, Koralewski P, Diaz-Rubio E, Rolland F, et al Open-label, uncontrolled, multicenter phase II study to evaluate the efficacy and toxicity of cetuximab as a single agent in patients with recurrent and/or metastatic squamous cell carcinoma of the head and neck who failed to respond to platinum-based therapy. Journal of clinical oncology: official journal of the American Society of Clinical Oncology. 2007;25(16):2171–7. Epub 2007/06/01. doi: 10.1200/JCO.2006.06.7447 . 17538161 10.1200/JCO.2006.06.7447

[7] Vermorken JB, Mesia R, Rivera F, Remenar E, Kawecki A, Rottey S, et al Platinum-based chemotherapy plus cetuximab in head and neck cancer. N Engl J Med. 2008;359(11):1116–27. Epub 2008/09/12. doi: 10.1056/NEJMoa0802656 . 18784101 10.1056/NEJMoa0802656

[8] Ferris RL, Blumenschein G Jr., Fayette J, Guigay J, Colevas AD, Licitra L, et al Nivolumab for Recurrent Squamous-Cell Carcinoma of the Head and Neck. N Engl J Med. 2016 doi: 10.1056/NEJMoa1602252 . 27718784 10.1056/NEJMoa1602252 PMC5564292

[9] Seiwert TY, Burtness B, Mehra R, Weiss J, Berger R, Eder JP, et al Safety and clinical activity of pembrolizumab for treatment of recurrent or metastatic squamous cell carcinoma of the head and neck (KEYNOTE-012): an open-label, multicentre, phase 1b trial. The Lancet Oncology. 2016;17(7):956–65. doi: 10.1016/S1470-2045(16)30066-3 . 27247226 10.1016/S1470-2045(16)30066-3

[10] Rischin D, Young RJ, Fisher R, Fox SB, Le QT, Peters LJ, et al Prognostic significance of p16INK4A and human papillomavirus in patients with oropharyngeal cancer treated on TROG 02.02 phase III trial. Journal of clinical oncology: official journal of the American Society of Clinical Oncology. 2010;28(27):4142–8. Epub 2010/08/11. doi: 10.1200/JCO.2010.29.2904 ; PubMed Central PMCID: PMC2953971. 20697079 10.1200/JCO.2010.29.2904 PMC2953971

[11] Argiris A, Li S, Ghebremichael M, Egloff AM, Wang L, Forastiere AA, et al Prognostic significance of human papillomavirus in recurrent or metastatic head and neck cancer: an analysis of Eastern Cooperative Oncology Group trials. Annals of oncology: official journal of the European Society for Medical Oncology / ESMO. 2014;25(7):1410–6. Epub 2014/05/07. doi: 10.1093/annonc/mdu167 ; PubMed Central PMCID: PMC4071756. 24799460 10.1093/annonc/mdu167 PMC4071756

[12] Jalali MM, Heidarzadeh A, Zavarei MJ, Sarmast H. p53 overexpression impacts on the prognosis of laryngeal squamous cell carcinomas. Asian Pac J Cancer Prev. 2011;12(7):1731–4. . 22126554

[13] Ueda M, Shimada T, Goto Y, Tei K, Nakai S, Hisa Y, et al Expression of CC-chemokine receptor 7 (CCR7) and CXC-chemokine receptor 4 (CXCR4) in head and neck squamous cell carcinoma. Auris Nasus Larynx. 2010;37(4):488–95. doi: 10.1016/j.anl.2009.11.012 . 20036791 10.1016/j.anl.2009.11.012

[14] Bektas-Kayhan K, Unur M, Boy-Metin Z, Cakmakoglu B. MCP-1 and CCR2 gene variants in oral squamous cell carcinoma. Oral Dis. 2012;18(1):55–9. doi: 10.1111/j.1601-0825.2011.01843.x . 21883707 10.1111/j.1601-0825.2011.01843.x

[15] Ramsey S, Lamb GW, Aitchison M, McMillan DC. The longitudinal relationship between circulating concentrations of C-reactive protein, interleukin-6 and interleukin-10 in patients undergoing resection for renal cancer. British journal of cancer. 2006;95(8):1076–80. Epub 2006/09/28. doi: 10.1038/sj.bjc.6603387 ; PubMed Central PMCID: PMC2360708. 17003778 10.1038/sj.bjc.6603387 PMC2360708

[16] Purdue MP, Hofmann JN, Kemp TJ, Chaturvedi AK, Lan Q, Park JH, et al A prospective study of 67 serum immune and inflammation markers and risk of non-Hodgkin lymphoma. Blood. 2013;122(6):951–7. Epub 2013/07/03. doi: 10.1182/blood-2013-01-481077 ; PubMed Central PMCID: PMC3739038. 23814017 10.1182/blood-2013-01-481077 PMC3739038

[17] Stotz M, Gerger A, Eisner F, Szkandera J, Loibner H, Ress AL, et al Increased neutrophil-lymphocyte ratio is a poor prognostic factor in patients with primary operable and inoperable pancreatic cancer. British journal of cancer. 2013;109(2):416–21. Epub 2013/06/27. doi: 10.1038/bjc.2013.332 ; PubMed Central PMCID: PMC3721392. 23799847 10.1038/bjc.2013.332 PMC3721392

[18] Melchardt T, Troppan K, Weiss L, Hufnagl C, Neureiter D, Trankenschuh W, et al Independent Prognostic Value of Serum Markers in Diffuse Large B-Cell Lymphoma in the Era of the NCCN-IPI. J Natl Compr Canc Netw. 2015;13(12):1501–8. Epub 2015/12/15. . 26656519 10.6004/jnccn.2015.0178

[19] Troppan KT, Schlick K, Deutsch A, Melchardt T, Egle A, Stojakovic T, et al C-reactive protein level is a prognostic indicator for survival and improves the predictive ability of the R-IPI score in diffuse large B-cell lymphoma patients. British journal of cancer. 2014;111(1):55–60. Epub 2014/05/31. doi: 10.1038/bjc.2014.277 ; PubMed Central PMCID: PMC4090740. 24874478 10.1038/bjc.2014.277 PMC4090740

[20] Guo Y, Shi M, Yang A, Feng J, Zhu X, Choi YJ, et al Platinum-based chemotherapy plus cetuximab first-line for Asian patients with recurrent and/or metastatic squamous cell carcinoma of the head and neck: Results of an open-label, single-arm, multicenter trial. Head & neck. 2015;37(8):1081–7. Epub 2014/04/09. doi: 10.1002/hed.23707 . 24710768 10.1002/hed.23707

[21] de Mello RA, Geros S, Alves MP, Moreira F, Avezedo I, Dinis J. Cetuximab plus platinum-based chemotherapy in head and neck squamous cell carcinoma: a retrospective study in a single comprehensive European cancer institution. PloS one. 2014;9(2):e86697 Epub 2014/02/12. doi: 10.1371/journal.pone.0086697 ; PubMed Central PMCID: PMC3916324. 24516537 10.1371/journal.pone.0086697 PMC3916324

[22] Lynggaard CD, Therkildsen MH, Kristensen CA, Specht L. The EXTREME regimen for recurrent/metastatic head and neck squamous cell carcinoma (R/M HNSCC): treatment outcome in a single institution cohort. Acta Oncol. 2015;54(7):1071–5. Epub 2014/10/25. doi: 10.3109/0284186X.2014.964308 . 25342535 10.3109/0284186X.2014.964308

[23] Vermorken JB, Stohlmacher-Williams J, Davidenko I, Licitra L, Winquist E, Villanueva C, et al Cisplatin and fluorouracil with or without panitumumab in patients with recurrent or metastatic squamous-cell carcinoma of the head and neck (SPECTRUM): an open-label phase 3 randomised trial. The Lancet Oncology. 2013;14(8):697–710. Epub 2013/06/12. doi: 10.1016/S1470-2045(13)70181-5 . 23746666 10.1016/S1470-2045(13)70181-5

[24] Argiris A, Li Y, Forastiere A. Prognostic factors and long-term survivorship in patients with recurrent or metastatic carcinoma of the head and neck. Cancer. 2004;101(10):2222–9. Epub 2004/09/29. doi: 10.1002/cncr.20640 . 15452834 10.1002/cncr.20640

[25] Nakayama M, Tabuchi K, Hara A. Clinical utility of the modified Glasgow prognostic score in patients with advanced head and neck cancer. Head & neck. 2015;37(12):1745–9. Epub 2014/07/06. doi: 10.1002/hed.23823 . 24989115 10.1002/hed.23823

[26] Selzer E, Grah A, Heiduschka G, Kornek G, Thurnher D. Primary radiotherapy or postoperative radiotherapy in patients with head and neck cancer: Comparative analysis of inflammation-based prognostic scoring systems. Strahlentherapie und Onkologie: Organ der Deutschen Rontgengesellschaft [et al]. 2015;191(6):486–94. Epub 2015/01/15. doi: 10.1007/s00066-014-0803-1 . 25583136 10.1007/s00066-014-0803-1

[27] Millrud CR, Mansson Kvarnhammar A, Uddman R, Bjornsson S, Riesbeck K, Cardell LO. The activation pattern of blood leukocytes in head and neck squamous cell carcinoma is correlated to survival. PloS one. 2012;7(12):e51120 Epub 2012/12/20. doi: 10.1371/journal.pone.0051120 ; PubMed Central PMCID: PMC3519486. 23251433 10.1371/journal.pone.0051120 PMC3519486

[28] Khandavilli SD, Ceallaigh PO, Lloyd CJ, Whitaker R. Serum C-reactive protein as a prognostic indicator in patients with oral squamous cell carcinoma. Oral oncology. 2009;45(10):912–4. Epub 2009/06/09. doi: 10.1016/j.oraloncology.2009.03.015 . 19502100 10.1016/j.oraloncology.2009.03.015

[29] Perisanidis C, Kornek G, Poschl PW, Holzinger D, Pirklbauer K, Schopper C, et al High neutrophil-to-lymphocyte ratio is an independent marker of poor disease-specific survival in patients with oral cancer. Med Oncol. 2013;30(1):334 Epub 2013/01/08. doi: 10.1007/s12032-012-0334-5 . 23292862 10.1007/s12032-012-0334-5

[30] Fang HY, Huang XY, Chien HT, Chang JT, Liao CT, Huang JJ, et al Refining the role of preoperative C-reactive protein by neutrophil/lymphocyte ratio in oral cavity squamous cell carcinoma. The Laryngoscope. 2013;123(11):2690–9. Epub 2013/04/27. doi: 10.1002/lary.24105 . 23619955 10.1002/lary.24105

[31] Misiukiewicz K, Bonomi M, Demicco E, Posner M. Controversies and role of HPV16 in recurrent/metastatic squamous cell cancers of the head and neck. Annals of oncology: official journal of the European Society for Medical Oncology / ESMO. 2014;25(8):1667–8. Epub 2014/05/31. doi: 10.1093/annonc/mdu194 . 24875798 10.1093/annonc/mdu194

[32] Lassen P, Eriksen JG, Hamilton-Dutoit S, Tramm T, Alsner J, Overgaard J. Effect of HPV-associated p16INK4A expression on response to radiotherapy and survival in squamous cell carcinoma of the head and neck. Journal of clinical oncology: official journal of the American Society of Clinical Oncology. 2009;27(12):1992–8. Epub 2009/03/18. doi: 10.1200/JCO.2008.20.2853 . 19289615 10.1200/JCO.2008.20.2853

